# Relative costs and benefits of alternative reproductive phenotypes at different temperatures – genotype-by-environment interactions in a sexually selected trait

**DOI:** 10.1186/s12862-018-1226-x

**Published:** 2018-07-11

**Authors:** Agata Plesnar-Bielak, Anna Maria Skwierzyńska, Kasper Hlebowicz, Jacek Radwan

**Affiliations:** 1 Institute of Environmental Sciences, Jagiellonian University, Gronostajowa 7, 30-387 Kraków, Poland; 2 Evolutionary Biology Group, Faculty of Biology, Adam Mickiewicz University, Poznań, Poland

**Keywords:** Sexually selected traits, Genetic variation, Genotype-by-environment interaction, Sexually selected phenotypes, Male morphs, Environmental conditions

## Abstract

**Background:**

The maintenance of considerable genetic variation in sexually selected traits (SSTs) is puzzling given directional selection expected to act on these traits. A possible explanation is the existence of a genotype-by-environment (GxE) interaction for fitness, by which elaborate SSTs are favored in some environments but selected against in others. In the current study, we look for such interactions for fitness-related traits in the bulb mite, a male-dimorphic species with discontinuous expression of a heritable SST in the form of enlarged legs that are used as weapons.

**Results:**

We show that evolution at 18 °C resulted in populations with a higher prevalence of this SST compared to evolution at 24 °C. We further demonstrate that temperature modified male reproductive success in a way that was consistent with these changes. There was a genotype-by-environment interaction for reproductive success – at 18 °C the relative reproductive success of armored males competing with unarmored ones was higher than at the moderate temperature of 24 °C. However, male morph did not have interactive effects with temperature with respect to other life history traits (development time and longevity).

**Conclusions:**

A male genotype that is associated with the expression of a SST interacted with temperature in determining male reproductive success. This interaction caused an elaborate SST to evolve in different directions (more or less prevalent) depending on the thermal environment. The implication of this finding is that seasonal temperature fluctuations have the potential to maintain male polymorphism within populations. Furthermore, spatial heterogeneity in thermal conditions may cause differences among populations in SST selection. This could potentially cause selection against male immigrants from populations in different environments and thus strengthen barriers to gene flow.

## Background

Sexual selection arises from reproductive competition and often leads to adaptation in the form of elaborated sexually selected traits (SSTs), which can serve as weapons in intra-sexual competition or as attractants to the opposite sex [1, 2]. Strong sexual selection should lead to a reduction of genetic variation in SSTs [3, 4]. Despite this, considerable within-sex genetic variation in SSTs seems to be the rule [5–7]. The nature of this variation has important consequences for processes such as the evolution of mating preferences [3, 8], ecological adaptation [9], and speciation [10, 11], but with few exceptions (eg. [12–15]) the mechanisms that maintain it are not well understood (see [16] for a review of potential mechanisms). One potentially important mechanism is a gene-by-environment (GxE) interaction for fitness, whereby genotypes associated with elaborate SSTs are favored under certain environmental conditions, but selected against under others [17–19]. To date, though, only a limited number of studies have demonstrated GxE interactions for male reproductive performance [20, 21] or attractiveness [22] but see [23], and only a few have shown GxE interactions that involve SSTs [24–27].

**Fig. 1:**
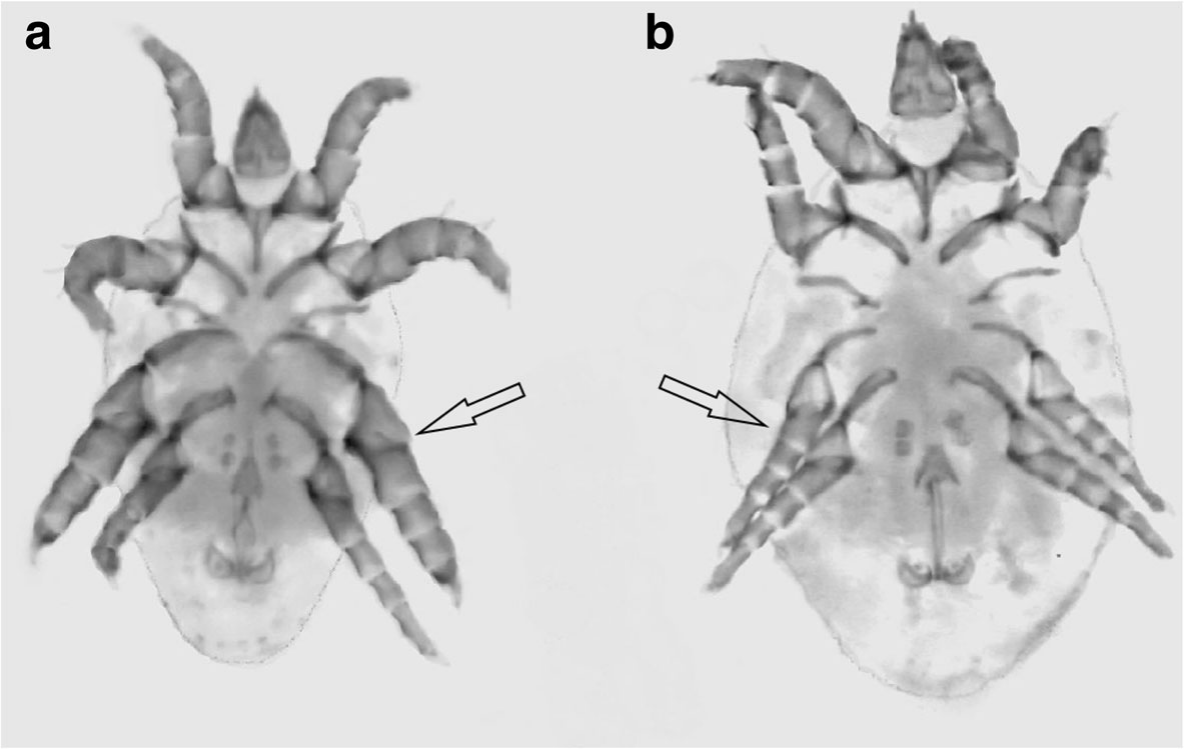
Abdominal view of fighter (**a**) and scrambler (**b**) male of the bulb mite (*Rhizoglyphus robini*). The main morphological difference between the morphs (shown with arrows) is that fighters have thickened, sharply terminated third pair of legs, which they use as weapons, while scramblers have unmodified female-like legs

Here, we tested whether genotypes that influence the expression of an elaborate SST interact with the thermal environment in determining fitness-related traits in males of the bulb mite (*Rhizoglyphus robini*) – a promiscuous acarid mite inhabiting underground parts of plants such as garlic, onion or leek. In this species, aggressive armored fighter males (Fig. 1a) possess a sharply terminated third pair of legs (the SST) that are used in fights with other males [28, 29]. Fighter males usually coexist with benign scrambler males, which have unmodified, female-like legs (Fig. 1b), and, unlike in some other acarids, morph frequencies are not affected by population density [30]. Instead, the expression of thickened legs is heritable [30, 31], but it is not clear how the genetic variation for male morph expression is maintained [32–34]. One possible explanation is that changes in temperature affect the strength of selection for thickened legs. Indeed, it has been reported that increased temperatures (28 °C) favor the evolution of monomorphic populations which consist solely of scramblers and females, a pattern that may be associated with temperature-dependent variation in the metabolic costs of particular phenotypes and their associated behavioral strategies [35]. Aggressive behavior, especially fighting, consumes a great deal of energy [36–38], such that its metabolic cost is likely to increase with temperature [39]. The link between the expression of the fighter phenotype and energy metabolism in the bulb mite has been further supported by a recent transcriptomic study which showed that many of the genes that are sex-biased in fighter males (but not in scramblers) are related to metabolism [40]. This suggests that the relative costs of expressing a particular morph may vary with temperature. If the expression of the fighter phenotype incurs lower costs at low temperatures and higher costs at high temperatures, genetic variation in SST expression may be maintained by seasonal temperature-induced fluctuations in the direction of selection on male morph (the mites reproduce continuously, with overlapping generations and several to a dozen or so generations per year).

**Fig. 2:**
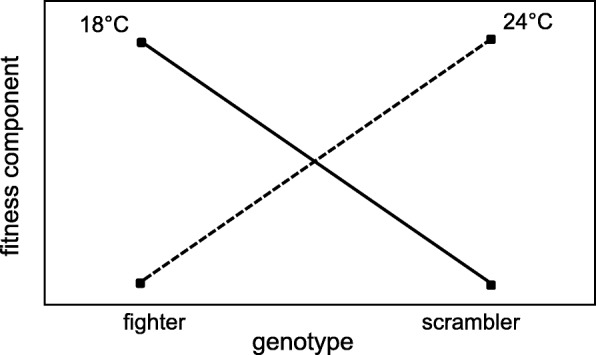
Schematic representation of possible shape of genotype-by-environment interactions expected if temperature modifies fitness of genotypes determining male morphs. We predicted that for at least some fitness components measured (male reproductive success, development time, longevity), fighter genotypes will have higher fitness at decreased temperature (18 °C), compared to standard laboratory temperature (24 °C), whereas for scramblers the difference should be less pronounced or even reversed. A cross-over relationship shown in the figure is just one possible shape of such interaction. Crossing of fitness functions, coupled with seasonal temperature fluctuations, could contribute to maintenance of genetic polymorphism for male morphs

In the current study, we investigated if low temperature favors the fighter phenotype in two independent experiments. Firstly, we complemented earlier results showing that high temperature (28 °C) leads to a decrease in fighter frequency compared to the standard laboratory temperature (24 °C) [35]. Here, we used experimental evolution to investigate whether decreased temperature (18 °C) leads to an increase in fighter frequency compared to 24 °C. Secondly, we investigated how temperature affects the fitness of male morphs. For that purpose, we measured the relative reproductive success of fighter males that competed with scramblers, as well as development time and longevity of both morphs at 18 °C and 24 °C. If thermal conditions affect the relative fitness of the male SST-determining genotypes, it would be reflected in a GxE interaction for the measured fitness traits (Fig. 2).

## Methods

### Morph frequencies at decreased temperature

We created experimental populations that differed in the initial proportions of male morphs; half of these populations evolved at decreased temperature (18 °C) and the other half at the control temperature (24 °C). We then observed how morph frequencies changed after 14 generations of evolution.

To establish experimental populations that differed in fighter proportions we used individuals from the 47th generation of replicate lines selected towards either a high frequency of scramblers (the S lines) or fighters (the F lines). These populations are nearly monomorphic, with the proportion of fighters reaching over 90% in the F lines and ca. 10% in the S lines. Details of the selection regime are described in [34]. Having male morphs nearly-fixed by selection, we could assume that these populations are enriched for alleles that affect fighter/scrambler expression. Although females do not express the fighter phenotype, they still carry genes that affect male morphs. By utilizing selection lines in which male morphs were almost fixed, we had control over the allele frequencies of these genes.

The evolution of morph frequencies might conceivably depend on the initial proportion in the population [41], so we decided to start our experimental evolution from two different proportions of fighters: 0.5 and 0.94. These were labeled “low” and “high” populations because these two proportions were lower and higher, respectively, than the proportion of fighters reported in standard laboratory conditions (0.69–0.73 at 24 °C; [32]). To establish the “high” populations, we took 17 fighters and 17 virgin females from each of the four F lines and one scrambler and one virgin female from each of the four S lines; in total, each “high” population consisted of 68 fighters, 4 scramblers, and 72 females. For the “low” populations we took nine fighters and nine virgin females from each F line and the same number of individuals from the S lines; each population consisted of 36 fighters, 36 scramblers, and 72 females.

We established 10 “high” populations and 10 “low” populations, which were then assigned to one of the two temperature regimes: decreased temperature (18 °C) or control temperature (24 °C). The control temperature was that to which both the source F and S lines, and our laboratory stock population, had been adapted. There were thus five populations in each treatment combination. Populations were kept in 4-cm-diameter plastic containers with water-soaked paper towels to maintain high humidity, and they were fed powdered yeast ad libitum.

Adults in all replicate treatments were allowed to interact and lay eggs for 5 days and then were removed from the containers. After 90–100% of the next generation reached the nymphal stage, 150 immature individuals from each population were transferred to a new container to start a new generation. We did not control morph proportion or sex ratio, instead allowing them to evolve freely.

After 14 generations of experimental evolution, we assessed morph proportions. To investigate the environmental effects of developmental temperature on morph expression, each population was tested at both 18 °C and 24 °C. We isolated 200 previously mated females from each experimental population, then transferred half of these to a new container at 18 °C and the other half to a new container at 24 °C. Females laid eggs for 3 days, after which they were removed. We collected 300 nymphs from each container and placed them individually in 0.8-cm-diameter glass tubes to avoid mortality due to aggression between males. After the adults emerged, we sexed them and recorded the morphs of the males.

### Looking for GxE interactions for fitness

#### a) Reproductive success

Reproductive success was measured for males from the stock culture using the ‘sterile male’ technique [42–44]. Our assay measured the relative reproductive success of fighter males competing with scrambler males.

To obtain experimental males whose parents had developed and mated at the experimental temperatures (18 °C and 24 °C), we placed 100 previously mated females from the stock culture in each of two containers and allowed them to lay eggs for 3 days. After this time, the females were removed, and one container was placed at 24 °C and the other at 18 °C. The eggs were left to develop. Approximately 3 days after the adults emerged and had an opportunity to mate, we transferred ca. 100 mated females per container to a fresh container and allowed them to lay eggs for 3 days, after which they were removed. When trithonymphs (last nymphal stage of the mites) emerged, they were isolated into individual vials and left until adulthood.

The emerged males were then used in the reproductive success assay. For that purpose, half of the males of each morph were irradiated with 20 kRad (200 Gy) of gamma radiation from Co_60_. After irradiation with such a dose, males are still able to fertilize eggs, but the eggs do not hatch [44]. Each irradiated male was placed in a glass tube with a non-irradiated male of the other morph and a female (both originating from the same experimental temperature) for 3 days. After this time, the adults were discarded. Thirty eggs from each vial (or, if there were less than 30, all the eggs that a female laid) were placed into a fresh tube at the standard temperature (24 °C). A week later, we calculated a fighter male’s reproductive success as the proportion of eggs that did not hatch (if the irradiated male was a fighter) or the proportion of eggs that hatched (if the irradiated male was a scrambler). Cases in which a female was found dead or did not lay any eggs were excluded. We also noted the morph of any males that died before the end of the experiment.

This procedure was a composite measure of reproductive success that combined both direct competition between males for the access to females and sperm competition.

#### b) Development time and longevity

We measured development time and longevity of fighter and scrambler males at 18 °C and 24 °C. The parents of these males came from the stock culture, but had developed and mated at the temperature at which male development time and longevity were measured (i.e. either 18 °C or 24 °C). The procedure was performed in three replicates for the lower temperature (18 °C) and in two replicates for the standard temperature (24 °C).

To obtain the parental generation, 120 previously mated females per replicate were taken from the stock culture and placed in a common container to lay eggs for 3 days at 24 °C. Following this, the females were discarded and the container with eggs was placed in an incubator at one of the two experimental temperatures. It was left there until all adults emerged and mated (20 days). Then, 40 mated females were placed in fresh containers and allowed to lay eggs for 24 h; the females were then removed and the eggs were left for further development. After the first trithonymphs were observed, the container was inspected daily for emerging males. For each male, we noted the morph and the date of emergence in order to calculate development time. Emerging females were discarded. To measure morph longevity, each emerging male was removed from the container and placed in a separate glass vial, which was inspected daily until the male’s death. During this time food was replenished when necessary to sustain ad libitum conditions and high humidity was maintained (> 90%). Male lifespan was recorded as the number of days from emergence until death (i.e. the period spent as an adult male).

### Statistics

Male morph frequencies in populations that underwent experimental evolution were analyzed using a generalized linear mixed-effect model with a binomial distribution of errors. Male morph occurrence was treated as binomial response variable (fighters were coded with 1 and scramblers with 0). The model included selection temperature, test temperature, initial fighter proportion (either high or low), and their interactions, all treated as fixed factors. Population identity nested in temperature of selection was treated as a random factor. Interactions were removed if non-significant and the fit of the models with and without a given interaction was compared. The simpler model was retained unless removing the interaction resulted in a significant decrease in goodness of fit, as measured using the ‘anova’ function in R [45].

Fighter males’ reproductive success was arcsine-transformed and used as a response variable in a linear model that included the fixed factors of temperature, the morph of the irradiated male, and their interaction. The numbers of fighters and scramblers that died during the experiment were compared separately for each temperature using Fisher’s exact test.

Development time was analyzed using a general linear mixed model with temperature, male morph, and their interaction as fixed factors and replicate nested in temperature as a random factor.

Due to the high heterogeneity of the longevity data, we used a general linear model that included the variance function, allowing to take into account heteroscedasticity of the data. Temperature, morph, and their interaction were included in the models as fixed factors, and square-rooted longevity was a response variable. Replicate was not included in the analysis as the results from the general linear model on square-rooted longevity (with temperature, morph, and their interaction treated as fixed factors and replicate as a random factor) showed that the effect of replicate explained only about 1% of the residual variance and was non-significant. To account for variance heterogeneity in our data, we first fitted a standard linear model with a homogenous variance structure (model zero) and used this for comparisons with subsequent models. Then, we fitted three additional models: model 1 allowed for differences in variances among temperatures, model 2 allowed for differences in variances between morphs, and model 3 allowed for different variances among all four combinations of temperature and morph. The models were built using the ‘gls’ function from the nlme package in R [46] and used VarIdent – a variance structure that allows variance to differ among levels of nominal variables [47]. Each of the models was then compared to the zero model using the log-likelihood ratio test. All three models were significantly better than the zero model, so they were ranked using the Akaike information criterion (AIC). The results of model 3, which yielded the lowest AIC value, are reported in the Results section. All analyses were performed in R 3.4.1 [48].

## Results

### Evolution of morph frequency

**Fig. 3:**
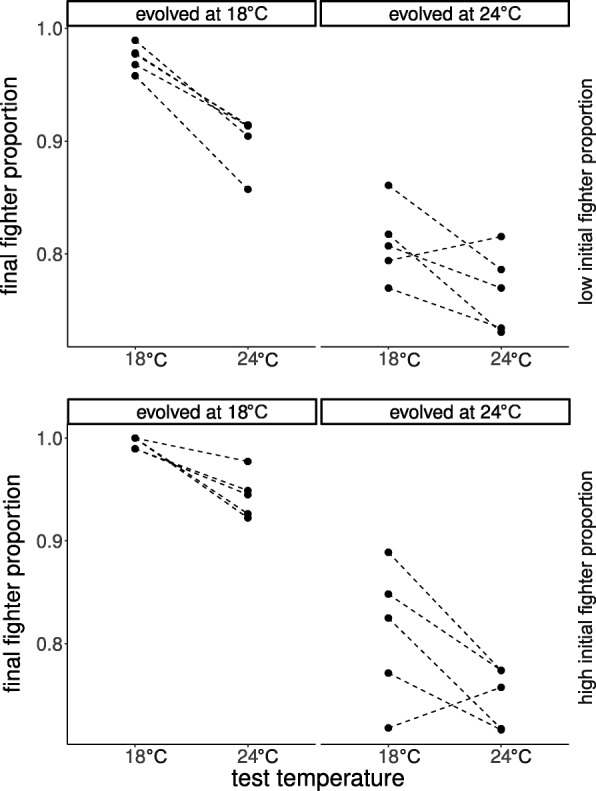
Proportions of fighters in populations following evolution at 18 °C or 24 °C and one generation of common-garden rearing at test temperatures of 18 °C or 24 °C. Experimental evolution was conducted with populations in which the initial proportion of fighters was either high (0.94) or low (0.5). Raw data are presented. Lines connect measurements from a given experimental population

After fourteen generations of evolution, lines that evolved at decreased temperature (18 °C) had higher proportions of fighters than lines that evolved at the standard temperature (24 °C) (F_1,18_ = 122.689, *P* < 0.001; Fig. 3). Morph frequencies were likewise affected by the test temperature used for the development of the final generation (F_1,4604_ = 23.889, *P* < 0.001); the lower temperature (18 °C) increased the proportion of fighters in both selection treatments. There was, however, a significant interaction between the temperature of selection and test temperature (F_1,4604_ = 20.293, *P* < 0.001) – the difference between selection treatments was greater when the final generation of mites developed at 18 °C compared to 24 °C (Fig. 3). We also found a significant interaction between selection temperature and initial fighter proportion (F_1,18_ = 9.689, *P* = 0.006), although the main effect of initial fighter proportion was insignificant (F_1,18_ = 0.726, *P* = 0.405). To further explore this interaction we performed separate analyses for both selection temperatures, and found that initial fighter proportion affected the final morph frequencies of populations that evolved at decreased temperature (F_1,8_ = 10.870, *P* = 0.011) but not at the control temperature (F_1,8_ = 0.306, *P* = 0.595).

### Reproductive success

**Fig. 4:**
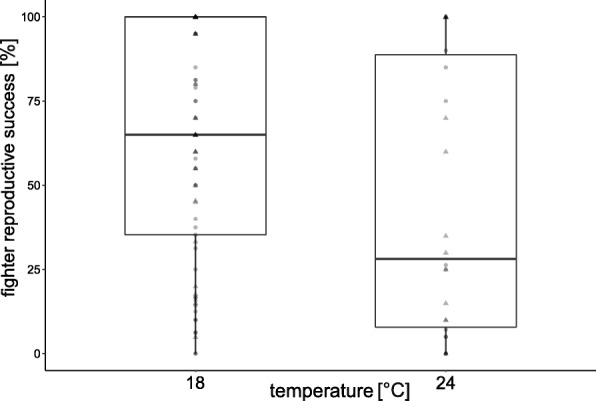
Reproductive success of fighters (percentage of eggs sired by a fighter when competing with a scrambler) in competition with scramblers measured at either 18 °C or at 24 °C (raw data and boxplots). Circles denote observations in which the fighter was irradiated and triangles denote observations in which the scrambler was irradiated

Fighters won reproductive competition with scramblers at 18 °C, but at 24 °C the situation was reversed (F_1,95_ = 7.657, *P* = 0.007, Fig. 4). There was also a significant negative effect of irradiation (F_1,95_ = 12.48, *P* < 0.001), but the temperature-by-irradiation interaction was non-significant (F_1,95_ = 0.004, *P* = 0.95).

At 18 °C, 27.4% of scramblers were found dead at the end of the experiment (17.6% in replicates in which the fighter was irradiated and 35.9% in replicates in which the scrambler was irradiated) compared to 2.7% of fighters (2.9% in replicates in which the fighter was irradiated and 2.6% in replicates in which the scrambler was irradiated). At 24 °C the percentages of males that died were 19.2% for scramblers (33.3% in replicates in which fighter was irradiated and 11.3% in replicates in which the scrambler was irradiated) and 15.4% for fighters (8.3% in replicates in which the fighter was irradiated and 21.4% in replicates in which the scrambler was irradiated). The difference between morphs in mortality was significant at 18 °C (Fisher’s exact test, *P* < 0.001), but not at 24 °C (Fisher’s exact test, *P* = 1).

### Development time

**Fig. 5:**
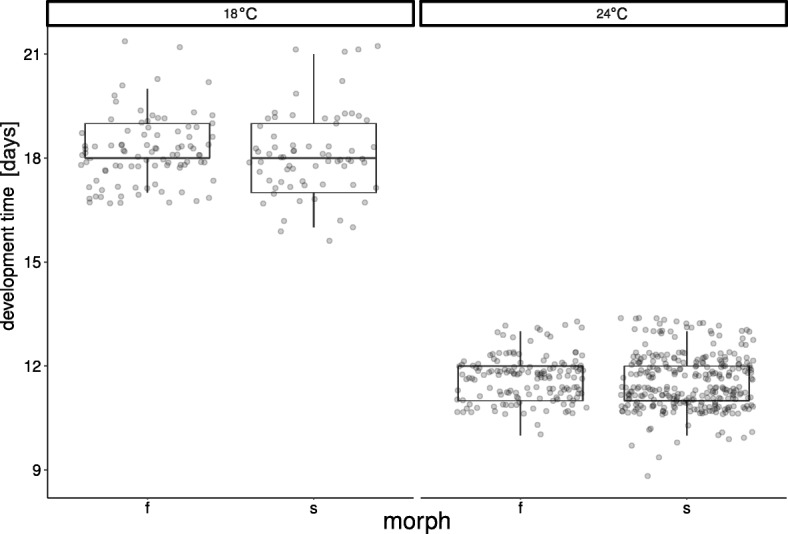
Developmental time of fighters (f) and scramblers (s) at 18 °C and 24 °C. Raw data and boxplots are shown

The differences between morphs in development time (F_1,509_ = 1.69, *P* < 0.194) and the temperature-by-morph interaction (F_1,509_ = 0.08, *P* = 0.777) were non-significant. However, decreased temperature significantly increased development time (F_1,4_ = 2169.99, *P* < 0.001, Fig. 5).

### Longevity

**Fig. 6:**
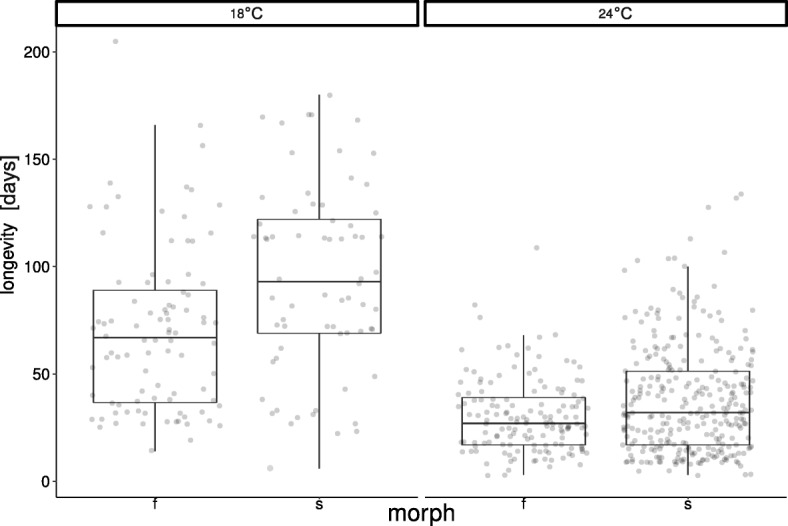
Longevity of fighters (f) and scramblers (s) at 18 °C and 24 °C. Raw data and boxplots are shown

Scramblers lived significantly longer than fighters (F_1,601_ = 5.715, *P* = 0.017), and the mites lived significantly longer at the lower temperature (F_1,601_ = 231.926, *P* < 0.001). The morph-by-temperature interaction was marginally non-significant (F_1,061_ = 3.675, *P* = 0.056), with a trend for the difference between morphs to be more pronounced at the lower temperature (Fig. 6).

## Discussion

In the current study, we show that the prevalence of fighter males with enlarged legs (an elaborate SST) in bulb mite populations evolves in response to the thermal environment. We further demonstrate that temperature modifies the relative competitive reproductive success of the two male morphs in a way that is consistent with the observed changes in morph frequencies. We discuss these findings in detail below. We also discuss the implications of our findings for the maintenance of polymorphism in male SST expression and for inter-population differentiation in SST expression, with possible consequences for ecological speciation.

### Temperature-dependent evolution of male morph frequencies

The proportion of fighter males in bulb mite populations has previously been shown to decrease across generations at increased temperature, leading to the evolution of monomorphic populations after ca. 30 generations at 28 °C [35]. This has led to the suggestion that the metabolic costs and/or benefits of expressing particular alternative reproductive phenotypes might change with temperature, which would thus drive changes in morph frequencies. In line with this hypothesis, in the current study we show that fighter frequency increased in populations that evolved at the lower temperature. This increase was observed irrespective of whether the initial proportion of fighters in the population was high or low, which suggests that this pattern is general rather than dependent on initial morph composition.

We also show here that the effects of developmental temperature on morph expression mimic those of evolutionary change: when a population develops at colder temperatures, it is more fighter-biased. One possible explanation for the observed plastic response to temperature involves temperature-dependent changes in body size. Indeed, bulb mites have been shown to follow the temperature-size rule, by which body size decreases with increasing temperature [35]; see [49] for more details on temperature-size rule. Furthermore, fighter males are known to develop from larger juveniles [50]. However, the same mechanism cannot explain the observed evolutionary change in morph frequencies, as temperature does not seem to affect body size evolution in this species (its effects on body size are purely environmental; [35]). Thus, decreased temperature must have led to the evolution of increased fighter frequency for reasons other than body size differences between thermal environments.

### GxE interactions for fitness-related traits and male morph expression

A likely reason why populations evolved to be fighter-biased at cold temperatures is that the relative success of fighters in male-male competition increases at decreased temperature (Fig. 4). In other words, there is a GxE interaction for male competitiveness which involves genes that determine the expression of the fighter morph (and hence SST). This might be a consequence of a decrease at lower temperatures in the cost associated with aggressiveness and fighting [39], and/or with the expression of the sexually selected trait itself [40, 51, 52]. The former possibility is supported by our observation of higher scrambler mortality, compared to fighters, at 18 °C but not at 24 °C. These difference is likely to be due to inter-male aggression, as our data show that, when inter-male competition was prevented, scramblers lived longer than fighters (as we further discuss below). Our findings thus suggest that fighters are more efficient in killing rivals at lower temperatures (scramblers have been shown to be unable to kill other males, so the mortality of fighters is either natural or a consequence of irradiation). It is also possible that temperature differentially affects sperm production or sperm competitiveness in different morphs, a hypothesis which remains to be investigated. Irrespective of the mechanism, however, we show here that the environment modified the fitness benefits of expressing SSTs and the associated fighter strategy. This finding is in line with other studies that demonstrate genotype-by-environment interactions in male reproductive performance (e.g., [20, 21]) and SSTs (e.g., [24–27]) in other species.

Interestingly, we did not find evidence that temperature differentially affected morph development time. This result was unexpected, as elaborate sexually selected traits are costly to develop [51] and such a cost should be temperature-sensitive [39]. On the other hand, this observation was consistent with a previous study showing that the morphs do not differ in development time at the standard temperature (24 °C; [50]). A similar result has been reported for another male-dimorphic acarid mite, *Sancassania berlesei* [53]. In *S. berlesei*, the investment necessary to develop thickened legs appears to occur at the cost of adult body size, rather than elongated development – fighters develop from heavier tritonymphs than scramblers, but morphs do not differ in adult mass [53]. Similarly, heavier tritonymphs have an increased probability of becoming fighters in *R. robini* [50], but another study reported no significant differences between male morphs in body mass [30]. It is likely that the development of thickened legs occurs at the cost of other structures [51, 54], a possibility worth pursuing in future research.

Consistent with a previous study [52], our results show that the physiological lifespan of scrambler males is longer than that of fighter males. This indicates that even when kept in isolation (with no interactions with females or other males), the expression of the fighter phenotype bears a life-history cost (although this cost is mitigated by age-related deterioration in male reproductive performance [52]). However, the longevity difference between morphs was not significantly modified by temperature. Thus, overall, we found that while temperature does not differentially affect the life histories of male morphs, it does strongly affect their competitive reproductive success, with the competitiveness of fighter males increasing at decreased temperature.

### GxE interactions and the maintenance of male morph polymorphism

The existence of GxE interactions for fitness is of key importance for sexual selection theory, as it creates the potential to maintain genetic variation in SSTs within populations exposed to conditions that vary in space [55] and/or time [56–58]. Our data show that the SST of the bulb mite (enlarged legs and the associated fighter behaviors) can be selected for at low temperatures, building on earlier work [35] that showed that it is selected against at high temperatures. Because they live on plant bulbs in the soil, bulb mites might experience quite substantial seasonal temperature fluctuations. Our results indicate that these fluctuations should cause selection on male morph expression to vary among seasons, potentially maintaining the genetic polymorphism underlying SST expression. Our present study shows that temperature-dependent change in the direction of selection acting on a male phenotypes occurs because temperature is a strong determinant of the reproductive competitiveness of male morphs. This effect could be further enhanced by correlative selection acting on male SSTs via females. For example, Skwierzyńska et al. (in prep) showed that females from fighter-selected lines (nearly fixed for fighter morph expression) were more fecund than those from scrambler-selected lines (nearly fixed for scrambler morph expression) when temperature was low, whereas at increased temperatures (28 °C), the female fitness ranking was reversed. Thus, selection acting via females should increase the overall effect of temperature on the frequencies of genes associated with alternative male morphs. In a fluctuating environment, then, this should increase the potential for the maintenance of genetic polymorphism (which is greater when fitness differences in alternative environments are high [59, 60]).

However, we note that temperature fluctuations do not explain why, despite the fact that fighters achieve higher reproductive success in mixed-morph populations at 22–26 °C [32], both morphs co-exist in a stable equilibrium at 24 °C [61]. Based on the results of [34] that females from fighter-selected populations had lower fecundity than those originating from populations selected for scrambler morphs, it has been speculated that intra-locus sexual conflict over the expression of (some of) the genes associated with male morphs is likely to maintain genetic polymorphism for the expression of enlarged legs ([34], Skwierzyńska et al. in prep.).

### GxE interactions and the potential for population differentiation with respect to SSTs

Irrespective of whether temperature fluctuations or intra-locus sexual conflict is primarily responsible for the co-existence of alternative male morphs in *R. robini* populations, temperature-based direct and indirect selection on morphs may cause inter-population differences in morph frequencies if populations inhabit different thermal environments. Little is known about such variation in nature, although a population devoid of fighters has been reported [62]. More generally, if environmental factors, as a rule, affect the costs and benefits of exaggerated sexual traits and their associated phenotypes, sexual selection may strengthen barriers to gene flow between populations occupying different environments. This is because developing the “wrong” sexually selected phenotype will likely increase the mismatch between genotype and environment unless the exaggerated sexual trait is entirely condition-dependent and migration takes place before the trait is developed [63]. In the bulb mite, however, temperature has only a moderate direct effect on this trait expression (Fig. 3), such that a migrant from a population adapted to a different temperature regime is likely to express a low-fitness morph in a new environment. Thus, in addition to mechanisms based on the condition-dependence of exaggerated SSTs [64], the strong genetic determination of such traits may also limit gene flow and facilitate ecological speciation.

## Conclusions

We demonstrate that the evolution of SST prevalence can be driven by environmental factors such as temperature. We also report the existence of a GxE interaction for male competitiveness that is probably responsible for these evolutionary changes. Thus, temperature-dependent selection on SSTs is a potential cause of differences in morph frequencies between populations. This suggests that selection for SSTs is likely to act differently in populations that occupy different environments, potentially strengthening barriers to gene flow between them. Furthermore, our results suggest that temperature fluctuations may change the direction of selection on the SST, increasing the potential for the maintenance of genetic variance for this trait within populations.

## Abbreviations

AIC: Akaike information criterion
GxE interaction: genotype-by-environment interaction
SST: sexually selected trait
